# Proficiency Testing of Metagenomics-Based Detection of Food-Borne Pathogens Using a Complex Artificial Sequencing Dataset

**DOI:** 10.3389/fmicb.2020.575377

**Published:** 2020-11-04

**Authors:** Dirk Höper, Josephine Grützke, Annika Brinkmann, Joël Mossong, Sébastien Matamoros, Richard J. Ellis, Carlus Deneke, Simon H. Tausch, Isabel Cuesta, Sara Monzón, Miguel Juliá, Thomas Nordahl Petersen, Rene S. Hendriksen, Sünje J. Pamp, Mikael Leijon, Mikhayil Hakhverdyan, Aaron M. Walsh, Paul D. Cotter, Lakshmi Chandrasekaran, Moon Y. F. Tay, Joergen Schlundt, Claudia Sala, Alessandra De Cesare, Andreas Nitsche, Martin Beer, Claudia Wylezich

**Affiliations:** ^1^Institute of Diagnostic Virology, Friedrich-Loeffler-Institut, Greifswald-Insel Riems, Germany; ^2^Department of Biological Safety, German Federal Institute for Risk Assessment, Berlin, Germany; ^3^Centre for Biological Threats and Special Pathogens, Robert Koch Institute, Berlin, Germany; ^4^Département de Microbiologie, Laboratoire National de Santé, Dudelange, Luxembourg; ^5^Department of Medical Microbiology, Amsterdam UMC University of Amsterdam, Amsterdam, Netherlands; ^6^Animal and Plant Health Agency, Addlestone, United Kingdom; ^7^Bioinformatics Unit, Institute of Health Carlos III (ISCIII), Madrid, Spain; ^8^Research Group for Genomic Epidemiology, National Food Institute, Technical University of Denmark, Lyngby, Denmark; ^9^Department of Microbiology, National Veterinary Institute (SVA), Uppsala, Sweden; ^10^Teagasc Food Research Centre, APC Microbiome Ireland and Vistamilk, Moorepark, Ireland; ^11^Nanyang Technological University Food Technology Centre (NAFTEC), Nanyang Technological University (NTU), Singapore, Singapore; ^12^Department of Physics and Astronomy, University of Bologna, Bologna, Italy; ^13^Department of Veterinary Medical Sciences, University of Bologna, Bologna, Italy

**Keywords:** background contamination, diagnostic assessment, high-throughput sequencing, metagenomics, pathogen, proficiency test, training

## Abstract

Metagenomics-based high-throughput sequencing (HTS) enables comprehensive detection of all species comprised in a sample with a single assay and is becoming a standard method for outbreak investigation. However, unlike real-time PCR or serological assays, HTS datasets generated for pathogen detection do not easily provide yes/no answers. Rather, results of the taxonomic read assignment need to be assessed by trained personnel to gain information thereof. Proficiency tests are important instruments of validation, harmonization, and standardization. Within the European Union funded project COMPARE [COllaborative Management Platform for detection and Analyses of (Re-) emerging and foodborne outbreaks in Europe], we conducted a proficiency test to scrutinize the ability to assess diagnostic metagenomics data. An artificial dataset resembling shotgun sequencing of RNA from a sample of contaminated trout was provided to 12 participants with the request to provide a table with per-read taxonomic assignments at species level and a report with a summary and assessment of their findings, considering different categories like pathogen, background, or contaminations. Analysis of the read assignment tables showed that the software used reliably classified the reads taxonomically overall. However, usage of incomplete reference databases or inappropriate data pre-processing caused difficulties. From the combination of the participants’ reports with their read assignments, we conclude that, although most species were detected, a number of important taxa were not or not correctly categorized. This implies that knowledge of and awareness for potentially dangerous species and contaminations need to be improved, hence, capacity building for the interpretation of diagnostic metagenomics datasets is necessary.

## Introduction

Approaches for the investigation of food-borne outbreaks regarding pathogen characterization, source attribution and risk assessment need to be precise, fast and independent from slow and biased cultivation techniques. Metagenomics-based high-throughput sequencing (HTS) is becoming a standard method for outbreak investigations of non-culturable, difficult-to-culture or slow-growing microorganisms (Koutsoumanis et al., 2019) yet protocols and analysis pipelines need to be standardized for routine use. In addition, training in result assessment and interpretation is needed for unexperienced users to be applicable as gold standard.

Problems with the analysis and the diagnostic assessment of HTS datasets may occur in several sample processing steps including sequencing, and during bioinformatics analysis. Beside the contamination of a sample during sampling and sample processing, microbial DNA can be introduced within the reagents during the preparation of sequencing libraries (Salter et al., 2014). Therefore, the specific reagent background should be known in ideal circumstances (Kirstahler et al., 2018; Wylezich et al., 2018) and at the very least, should be taken into consideration when assessing the taxa found in metagenomics datasets. Cross-contamination of multiplexed libraries is possible due to adapter swapping (Sinha et al., 2017) or carry-over between runs (Illumina, 2013; Höper et al., 2016). Furthermore, the interpreter of data should be aware of possible false-positives detected due to contaminated genomes and insufficiently curated databases (e.g., Kirstahler et al., 2018). All these points are very important when interpreting metagenomic datasets in search for possible pathogens that may be less abundant in terms of sequencing reads.

Some ring trials and proficiency tests have previously been conducted to push forward the standardization of HTS approaches and their implementation into clinical diagnostic routine and diagnosis reporting systems. Metagenomics-based proficiency tests for pathogen detection have often been focused on virus detection (Brinkmann et al., 2019; Junier et al., 2019; Zamperin et al., 2019). These studies highlighted that recognition of viruses that exhibit high mutation rates can cause some difficulties and might only be possible by inclusion of protein-based alignment approaches (Brinkmann et al., 2019). In addition, the undetected pathogen of relevance can also be a bacterial or parasite pathogen, which could be comprised in the sample but be masked by the sample background. Thus, interpreters need to train their capability recognizing the different categories of contaminations.

To some extent, the wet lab procedures for metagenomics-based pathogen detection were already standardized within the European Union funded COMPARE-network.^1^ Within this framework, a first metagenomics-based virus detection proficiency test with separate parts addressing bioinformatics only or sample processing combined with bioinformatics and result assessment, respectively, was conducted (Brinkmann et al., 2019). Due to the importance of foods in the transmission of zoonotic agents, a second proficiency test for metagenomics-based pathogen detection in food was organized in 2018 within the COMPARE network. Again, this proficiency test consisted of two independent parts addressing either the sample processing (wet lab) or bioinformatics combined with result assessment (dry lab). In the laboratory part (wet lab), the participants had to generate a metagenomic sequence dataset from a piece of smoked salmon that was spiked with a complex mock community (bacteria, fungi, eukaryotic parasite, and virus). The wet lab part was analyzed and is published separately in a companion manuscript (Sala et al., Unpublished). In the present study, the bioinformatics and assessment part (dry lab), the participants had to analyze a synthetic dataset and assess their obtained result. While here the analysis of the same dataset by all participants ensured the comparability of the submitted results, in the wet lab part, the comparison of the wet lab workflows was ensured by the centralized sequence data analysis. Moreover, participants were free to decide in which of the two independent parts they participated.

Whereas in the first COMPARE virus proficiency test (Brinkmann et al., 2019) the quality of the data analysis software was in the focus, in the present proficiency test we focused on the participants assessment of the results obtained by the software. Hence, the purpose was (i) testing the interpretation of results obtained from the software analysis by the participants, i.e., the recognition of potentially dangerous species and (ii) the awareness of artifacts occurring in the sample processing and sequencing.

## Materials and Methods

### Organization of the Dry Lab Proficiency Test

Like the COMPARE virus proficiency test (Brinkmann et al., 2019), the food metagenomics proficiency test dry lab part was initiated within the COMPARE network, and arranged by the network partner Friedrich-Loeffler-Institut between April and June 2018. The participants of the dry lab part of the food metagenomics proficiency test received a synthetically generated complex metagenomics sequencing dataset that they had to analyze and assess their obtained results regarding potentially present pathogens, sequencing artifacts, potential lab contaminations, and other information deemed relevant for assessment.

### Participants of the Dry Lab Part

Twelve participants applied for the COMPARE food metagenomics dry lab proficiency test and completed the survey. Participants were registered from Denmark (*n* = 1), Germany (*n* = 3), Luxembourg (*n* = 1), Ireland (*n* = 1), Italy (*n* = 1), Netherlands (*n* = 1), Singapore (*n* = 1), Spain (*n* = 1), Sweden (*n* = 1), and United Kingdom (*n* = 1). The 12 participants represented 12 different institutes or organizations. Information about the participants’ background is given in Table 2.

### Creating a Synthetic Metagenomics Dataset

The synthetic metagenomics dataset was created using ART_Illumina, Q Version 2.5.8 (Huang et al., 2012) and a number of EST (expressed sequence tag) and coding sequence data retrieved from the NCBI database (see Table 1). For details of the dataset creation, please refer to Supplementary Materials and Methods.

**TABLE 1:** Composition of the simulated sequence dataset.

Species	Super-Kingdom	Category	Rationale	Number Reads
*Bacteroides fragilis*	Bacteria	Opportunistic pathogen	Analogous to wet-lab proficiency test mock community	20,000
*Burkholderia pseudomallei*	Bacteria	Pathogen	Burkholderiaceae also found in real smoked salmon sample	8,000
*Escherichia coli*	Bacteria	Pathogen	Analogous to wet-lab proficiency test mock community, also found in real smoked salmon sample	80,000
*Salmonella enterica*	Bacteria	Pathogen	Analogous to wet-lab proficiency test mock community	125,000
*Fusobacterium nucleatum*	Bacteria	Opportunistic pathogen	Analogous to wet-lab proficiency test mock community	40,000

*Lactobacillus acidophilus*	Bacteria	Background	Food additive	175,000
*Lactobacillus delbrueckii*	Bacteria	Background		25,000

*Listeria monocytogenes*	Bacteria	Pathogen	Frequently found as food contamination	10,000
*Mycobacterium colombiense*	Bacteria	Opportunistic pathogen	Mycobacteria with increasing impact as food spoilage	2,000
*Pseudomonas libanensis*	Bacteria	Background	*P. libanensis* previously detected in food; Pseudomonadaceae also found in real smoked salmon sample	200

*Anisakis berlandi*	Eukaryota	Pathogen	Analogous to *Cryptosporidium parvum* from wet-lab proficiency test mock community	1,212
*Anisakis brevispiculata*	Eukaryota	Pathogen		246
*Anisakis paggiae*	Eukaryota	Pathogen		248
*Anisakis pegreffii*	Eukaryota	Pathogen		2,051
*Anisakis physeteris*	Eukaryota	Pathogen		262
*Anisakis simplex*	Eukaryota	Pathogen		6,044
*Anisakis typica*	Eukaryota	Pathogen		247

*Aspergillus flavus*	Eukaryota	Opportunistic pathogen	Analogous to *Saccharomyces cerevisiae* from wet-lab proficiency test mock community, toxin producer	5,000

*Danio rerio*	Eukaryota	Database misclassification		4,310
*Brugia malayi*	Eukaryota			64
*Caenorhabditis remanei*	Eukaryota			193
*Scomber japonicus*	Eukaryota			181

*Oncorhynchus mykiss*	Eukaryota	Food	*O. mykiss* EST data as host background	9,451,675
African swine fever virus (ASFV)	Viruses	Run contamination	DNA virus; barcode mis-alignment/index swapping on Illumina MiSeq	15
Norwalk virus	Viruses	Pathogen	RNA virus; typical food contaminant	946
Escherichia virus phiX174 (phiX174)	Viruses	Run contamination	Barcode mis-alignment/index swapping on Illumina MiSeq	735
Aspergillus foetidus dsRNA mycovirus (AfdsV)	Viruses	Background	Model for virus of the contaminant *Aspergillus flavus*	107
Sum				9,958,736

### Requirements to Participants

In parallel with the dry lab synthetic dataset, an email (see email 1 in the Supplementary Materials and Methods) was sent to all participants that included the necessary instructions for the data and assessment preparation and delivery. The requirements were later further specified (see email 2 in the Supplementary Materials and Methods). Briefly, the participants were asked to use their routine bioinformatics for taxonomic read classification and to submit a table in which the taxonomic classification for all reads was recorded in two columns (“Read accession” and “Species”). Hereinafter, this table is called “read assignment table.” Furthermore, they were asked to prepare a report as Word file or pdf containing their summary and assessment of the read classifications. They were asked to especially consider potentially present pathogens, sequencing artifacts, possible sequencing lab contaminations, and other facts they deemed important. This document is called “summary and assessment file” in the following.

The participants had about 50 days (25 April–15 June 2018) for conducting a bioinformatics analysis with the dataset provided and for assessing the results from obtained data until the delivery of their results.

### Data Analyses

The submitted read assignment tables were analyzed using R and RStudio (R v3.6.2, R Core Team, 2019; RStudio v1.2.5033).^2^ For an assessment of the taxonomic classifications reported in the participants’ read assignment tables, sensitivity, specificity, correct classification rate, positive predictive value, and negative predictive value were calculated from the read-to-taxon assignments. For further details of these calculations, please see Supplementary Materials and Methods. For the final rating of the participants’ assessments, the expected assessments were defined as shown in Table 1. The submitted assessments were rated in five different classes: (i) species detected and rated by the participant as expected, (ii) species detected and rated more serious than expected, (iii) species detected but assessed less critical than expected, (iv) species detected but not assessed, and (v) species not detected (according to the submitted read assignment table).

### Data Availability

The synthetic metagenomics dataset simulating a contaminated trout was submitted to European Nucleotide Archive and is accessible under the study accession number PRJEB37463. The read-accessions in this file comprise the database identifiers of the used reference sequence and the suffix “-fpt2018” with a continuous numbering per input sequence, for instance “lcl|NC_026023.1_cds_YP_009113336.1_1-fpt2018105”.

## Results and Discussion

The aim of the present proficiency test was in particular to test the interpretation of results obtained from metagenomics sequencing datasets based on the software analysis performed by the participants. For this purpose, the detection of potentially dangerous species was most important but also the awareness and recognition of artifacts that may occur during the wet lab sample processing and sequencing.

### General Considerations of the Provided Files

All participants except one (P10) uploaded the requested read assignment table with the read to species assignments output by their applied software pipeline, eight of which in due time, and three with a delay. All participants sent in a summary and assessment file of their obtained results (see Supplementary Material).

**FIGURE 1:**
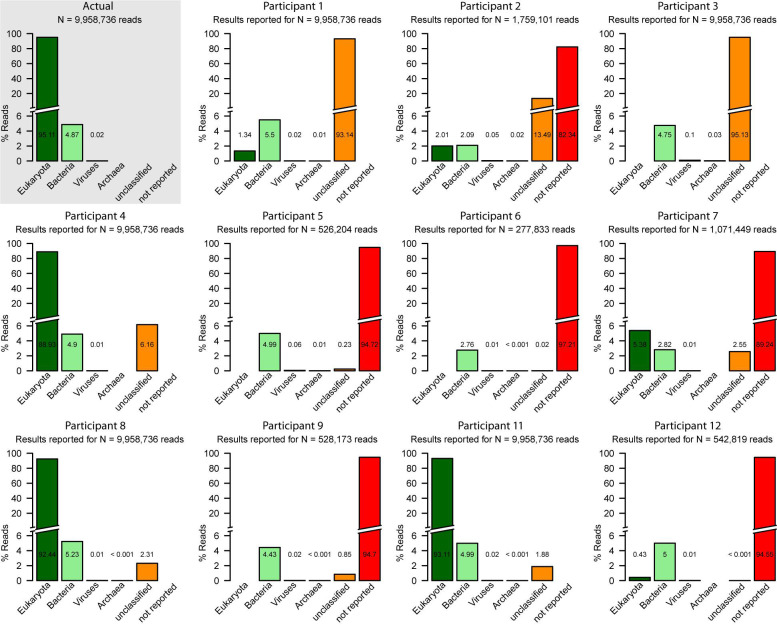
Summary of the read assignments at the superkingdom-level for 11 participants that provided the requested read assignment table with the read to species assignments (except P10). Only five read assignment tables (P1, P3, P4, P8, and P11) contained an assignment for all reads of the dataset. Only the compositions reported by P4, P8, and P11 fit the known actual composition (actual; upper left) of the dataset.

The format requested for the preparation of the read assignment tables (see above and in Supplementary Materials and Methods) turned out to be not clear for all participants. Six participants sent in incomplete tables, only reporting assignments for a small fraction of reads (see Figure 1). Moreover, only five participants sent in their read assignment tables in the requested format. In the remaining cases, participants reported more details with strain or isolate names or incomplete assignments only to a level between superkingdom and genus. Especially the users of Kraken did not report in the requested format but used the Kraken format including the full taxonomic path up to the most specific level reported. A similar observation was recently made by the organizers of another ring trial, who stated that the participants did not in all cases use official scientific names. They emphasized the importance of using a standardized set of species names based on NCBI taxonomy for reporting (Junier et al., 2019).

The summary and assessment files of four participants (P2, P4, P5, and P10) only contained the requested summary table without an assessment. The remaining eight participants provided both the requested summary table and an assessment of the reported results.

### Insights From Analysis of the Read Assignment Tables

Of the uploaded 11 read assignment tables, only five (from P1, P3, P4, P8, and P11) contained an assignment for all reads of the dataset; the remaining six contained assignments for approximately 3–18% of the reads (Figure 1). The reasons not to report assignments for the missing reads were (i) filtered low quality (P2, P6, P7, and P9), (ii) filtered eukaryotic sequences (P2, P5, P6, and P9), or (iii) no justification (P12). In two cases (P1 and P3; see Figure 1) the majority of the reads (approximately 95%) was reported “unclassified.” In one case (P1), this was partly due to incomplete classification to higher-level taxonomic entities only. Three participants (P4, P8, and P11; Figure 1) reported classifications for the majority of the reads and only in these cases the overall composition resembled the actual one (Figure 1).

**FIGURE 2:**
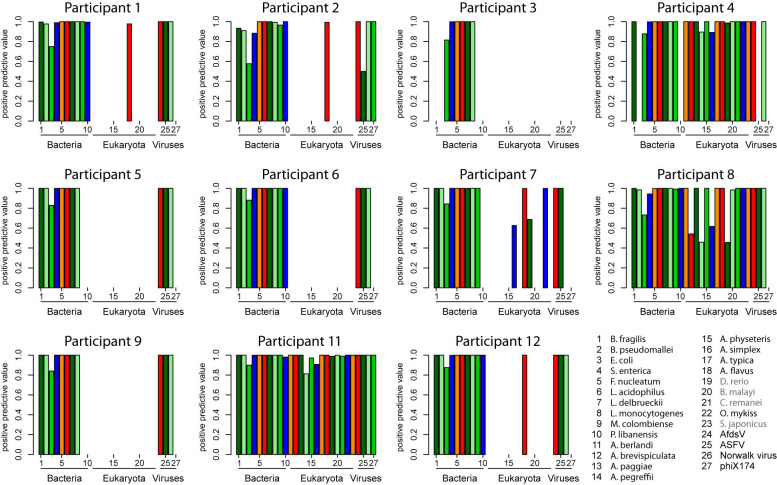
Positive predictive values of read assignments calculated from the complete read set, calculated based on the species assignments. Sequences of the taxa labeled gray (*Brugia malayi*, *Caenorhabditis remanei*, *Danio rerio*, and *Scomber japonicus*) were downloaded unintentionally as part of the *Anisakis* sequence dataset.

**FIGURE 3:**
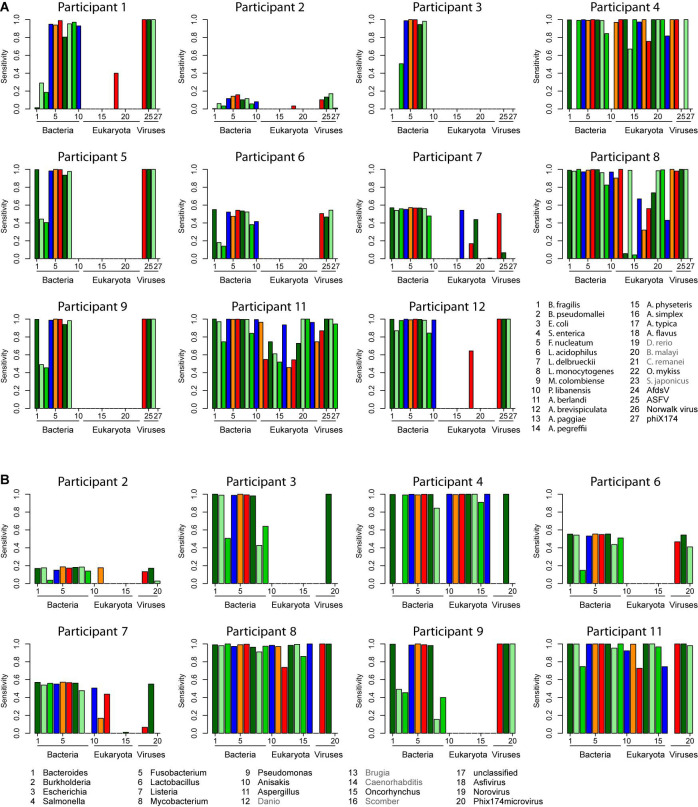
Sensitivity of read assignments calculated from the complete read set. **(A)** Sensitivities calculated based on the species assignments. **(B)** Sensitivities calculated based on the genus assignments. Sequences of the taxa labeled gray (*Brugia malayi*, *Caenorhabditis remanei*, *Danio rerio*, and *Scomber japonicus*) were downloaded unintentionally as part of the *Anisakis* sequence dataset.

The provided read assignments were used to calculate the key characteristics of the classifications. Namely, specificity, correct classification rate, and negative predictive values (Supplementary Figures 1–3), as well as positive predictive value (Figure 2) and sensitivity (Figure 3) were determined. While the specificity, correct classification rate (except for host sequences), and negative predictive value (except for host sequences) were in all analyses high, the positive predictive value (Figure 2) and especially the sensitivity (Figure 3) were in some cases insufficient. Both the positive predictive value and the sensitivity appear to be compromised by either the use of incomplete databases for the taxonomic classification and/or by improper pre-processing of the dataset (compare data analysis workflows and comments in Table 2). In three cases (P2, P6, and P7), pre-processing using software default or otherwise accepted parameters appears to have removed reads by chance, because the sensitivity is at the same low level for all detected species, meaning that this was not a species-specific effect (see results for participants P2, P6, and P7 in Figure 3). The high rate of discarded reads could have prevailed the respective participants to have a closer look at the reason for this phenomenon and modify the software settings depending on the dataset quality. The notion that this was not a species-specific effect is also emphasized by the high specificity mentioned above and by the unaltered sensitivity in case of calculation at the genus, instead the species level (see Figure 3B). If it was a species-specific effect, this should result in an improved sensitivity because then reads that are classified to closely related species should improve the result, as can indeed be seen by comparison of results obtained for participants P8 and P11 (compare Figure 3B). This was possibly the case with *Pseudomonas libanensis* in the results of participants P3, P4, P7, and P9 (Figure 3B).

**TABLE 2:** Participants, their background and applied data processing and important comments from their summaries.

Participants	Participants’ sector	Data processing workflow	Participants comments
P1	Food	– Quality assessment w/FastQC (Wingett and Andrews, 2018) – Quality trimming w/fastp (Chen et al., 2018) – Taxonomic classification w/Kraken (custom database and MiniKraken DB; Wood and Salzberg, 2014) – Additionally pathoLive analysis for classification of viral reads (Tausch et al., 2018)	– FastQC revealed bases of bad quality at the beginning of the reads. Therefore the reads were trimmed – kraken analysis with custom database resulted in many false-positive results; therefore, results were confirmed with BLASTn (Boratyn et al., 2012).
P2	Human	– Quality assessment w/FastQC (Wingett and Andrews, 2018) – Quality trimming w/Trimmomatic (Bolger et al., 2014) – Taxonomic classification based on mapping and assembly w/Pikavirus (in-house in-development tool at https://github.com/BU-ISCIII/PikaVirus) – Taxonomic classification based on mapping and assembly w/oases (Schulz et al., 2012) – Taxonomic classification based on rRNA clustering w/MeTRS (Cottier et al., 2018) – Taxonomic classification based on protein identity analysis w/Kaiju (Menzel et al., 2016)	– trimming parameters: nucleotides at 3′ with phred quality <10 or average quality ≤15 (window size 4), removal of reads shorter 50 bp – trimming dropped 8137323 sequences (81.71%) – unusual bad quality 5′ end was observed at the 25 firsts bases
P3	Human	– Taxonomic classification w/Kraken (Wood and Salzberg, 2014) as implemented on Galaxy public server	– Norovirus GV (murine norovirus, not a human pathogen) Unlikely to be on food sample – Hepatitis C virus (human pathogen, but route of transmission is via blood), highly unlikely to be found on food sample, and contamination with human blood?
P4	Food	– Quality assessment w/FastQC (Wingett and Andrews, 2018) – Quality/Adapter trimming w/BBduk v. 36.49 (https://sourceforge.net/projects/bbmap/) – Taxonomic classification w/MGmapper (Petersen et al., 2017)	– mapped against a phiX174 reference sequence to remove potential control library reads – Certain contaminations can be difficult to identify without control samples. For example, certain microorganisms may be part of the natural microbiome of fish or could have been introduced during sample handling and processing.
P5	Veterinary	– Quality assessment w/FastQC (Wingett and Andrews, 2018) – Quality/Adapter trimming w/Trimmomatic (Bolger et al., 2014) – Host sequence removal w/BWA-MEM (Li, 2013) – Taxonomic classification w/Kraken (MiniKraken database; ref. Wood and Salzberg, 2014)	– Filtered for minimum read count (threshold 500 reads) – species for which there were less than 1,000 reads would need further confirmation before release of the information – PhiX carry over from the sequencing lab
P6	Human	– Quality assessment w/FastQC (Wingett and Andrews, 2018) – Quality trimming w/Trimmomatic (Bolger et al., 2014) – Host sequence removal w/BBmap (id threshold 0.65; https://sourceforge.net/projects/bbmap/) – Taxonomic classification w/Kraken (database version 13/10/2017; Wood and Salzberg, 2014)	– FastQC result: No adapters detected
P7	Human	– Quality/Adapter trimming w/Trimmomatic (Bolger et al., 2014) – Host sequence removal w/Bowtie2 (Langmead and Salzberg, 2012) – Taxonomic classification w/MALT (Herbig et al., 2016), DIAMOND (Buchfink et al., 2015), MEGAN (Huson et al., 2016), custom database with refseq viruses, bacterial, fungi, and protists	– Pathogen of importance is Norovirus GV – Abundance of a cloning vector could be an artifact of sequencing reagents and preparation
P8	Veterinary	– Quality trimming w/RIEMS (Scheuch et al., 2015) – Taxonomic classification w/RIEMS (Scheuch et al., 2015); ncbi nt	– The Calicivirdae/Norwalk virus reads indicate the presence of noroviruses in the sample. This is the most important entero-pathogenic virus in the analyzed sample
P9	Food	– Species-level classification w/Kraken (Wood and Salzberg, 2014) – Multi-locus sequence types (MLSTs) reconstructed w/MetaMLST (Zolfo et al., 2017) – Strain-level identification w/PanPhlAn (Scholz et al., 2016)	None
P10	Food	– Quality assessment w/FastQC (Wingett and Andrews, 2018) – Quality trimming w/cutadapt (part of MGmapper processing; (Martin, 2011) – Taxonomic classification w/MGmapper (Petersen et al., 2017)	– no hits w/default settings, re-analysis w/adjusted parameters (max mismatch ratio = 0.15, min read count = 20) – sequence GC content measured by FastQC is reported as failure
P11	Veterinary	– Host sequences were removed by blasting (BLASTn) against a database created from the *Oncorhynchus mykiss* isolate Swanson WGS data (NCBI acc. MSJN00000000.1) using an E-value cutoff 1E-100 – Taxonomic classification w/carried out by blasting (BLASTn) the remaining reads against an NCBI nt database using TimeLogic^®^ DeCypher^®^ server (Active Motif Inc., Carlsbad, CA, United States) with an E-value cutoff of 1E-5. The assignment of sequences to species were carried out by an in-house Python script using the nucl_gb.accessions2taxid (accession to taxid) and names.dmp (taxid to scientific names) files available from the resources at NCBI	– We included Murine norovirus in the table despite it is not human pathogen
P12	Veterinary/Food	– Quality assessment w/FastQC (Wingett and Andrews, 2018) – Quality/Adapter trimming w/cutadapt (part of MGmapper processing) (Martin, 2011) Taxonomic classification w/MGmapper (Databases: Bacteria, Bacteria_draft, Human Microbiome, Virus, Fungi, Protozoa, and MetaHitAssembly) (Petersen et al., 2017)	None

**FIGURE 4:**
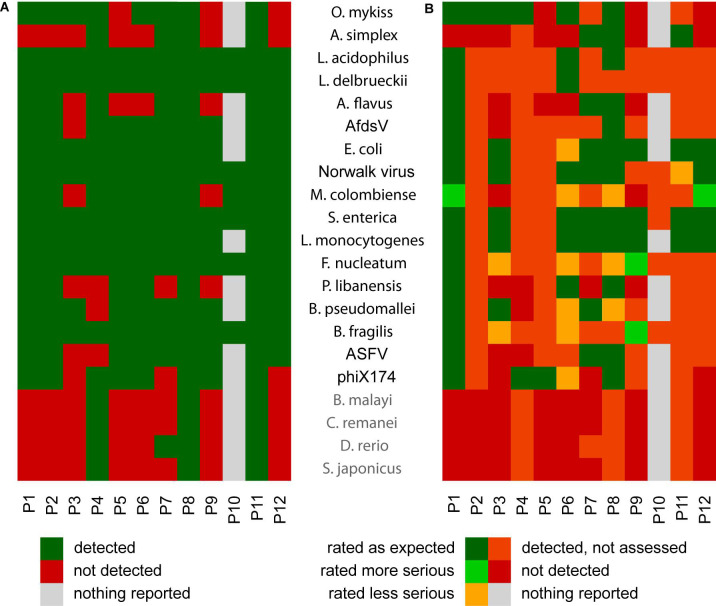
Summary of the read-assignment tables **(A)**, and assessment and interpretation of the assignments **(B)**. Heatmap showing the positive (comprised species detected; green) and negative (comprised species NOT detected; red) results of the software analyses. The results shown for participants are based on their uploaded read assignment tables, except for P10, for which the results are derived from their summary table and assessment **(A)**. Heatmap summarizing assessments of the detected species by the participants **(B)**. Sequences of the taxa labeled gray (*Brugia malayi*, *Caenorhabditis remanei*, *Danio rerio*, and *Scomber japonicus*) were downloaded unintentionally as part of the *Anisakis* sequence dataset.

Despite the partly suboptimal results calculated from the submitted complete classifications, Figure 4A clearly demonstrates that in most cases the majority of the comprised species were detected by the used software. A marked exception were those species that were not deliberately included in the dataset, namely *Scomber japonicus*, *Brugia malayi*, *Danio rerio*, and *Caenorhabditis remanei*. These were only detected by three participants (P4, P8, and P11) who used (nearly) complete databases for the taxonomic classification. All other participants reported to have used custom databases or the MiniKraken database that also comprises only selected sequences (Wood and Salzberg, 2014). This database effect is also highlighted by the fact that at least seven and five participants, respectively, failed to detect *Anisakis simplex* and *Aspergillus flavus*, two eukaryotic taxa. This emphasizes the impact of the database used for taxonomic classification to obtain a comprehensive classification. This result is in contrast to the results of another proficiency test (Junier et al., 2019), where the impact of the database was negligible compared with the influence of the applied algorithms.

### Insights From Evaluations of Participants’ Summary and Assessment Files

In the present proficiency test, the assessment of the results turned out to be the most critical part. Overall, the quality of the reports was varying. Although explicitly requested, the results of the taxonomic binning were not in all cases assessed regarding the requested categories (potentially present pathogens, sequencing artifacts, possible sequencing lab contaminations, and other important facts). Looking at the summary and assessment files, there was no correlation between the overall quality of the assessment and the background of the participant (compare Table 2). Though most species were detected (Figure 4A), the overall result was suboptimal (see summary in Figure 4B). For this assessment, the participants’ assessments were compared with the expected categorization of the respective species (compare Table 1). The reasons for the observed deviations may be diverse, located both at the technical level and at the individual experiences of the personnel.

At the technical level, unsuitable parametrization of the analysis may be a possible cause for missing important species from the result the diagnostician gets for the assessment, i.e., arbitrary thresholds for taking detected species into consideration. For instance, thresholds set for read numbers assigned to a single species can prevent detection, as was the case with P5 (reporting a minimum read number of 500 for each individual species) failing to recognize the African swine fever virus (15 reads) and the Aspergillus foetidus dsRNA mycovirus (107 reads). Moreover, minimum genome coverage of detected species, or too stringent cut-offs for the identity of reads with reference sequences may prevent species from appearing in the table of the detected species. Another technical issue was the use of incomplete databases for taxonomic binning (see above).

With regard to the individual experience of the personnel, a number of different reasons could be considered. In two cases (P3 and P11), the assessment of Norovirus was based on the detected viral strain, which was closest to murine strains and therefore assessed as “no human pathogen”. However, indications of potential pathogens should always be followed up, because of the possibility of detecting a modified or novel pathogen with only weak relationship with the closest known relative in the database. In other cases, assessment of the software output by personnel not trained for this task (e.g., assessment by bioinformaticians instead of microbiologists, physicians, or veterinarians), insufficient awareness of the impact of certain species due to insufficient training, or maybe unsatisfactory consideration due to time constraints may have caused the result.

## Conclusion

The dry-lab part of this ring-trial showed that despite the abovementioned shortcomings in some analyses (namely usage of incomplete databases or unsuitable data pre-processing), overall the used software appears to have matured over the last years to allow for the correct identification of the majority of organisms represented in a metagenomics dataset. However, for a truly beneficial effect of diagnostic metagenomics for the detection of potentially present pathogens, it is especially necessary to put more effort into the training for the assessment and interpretation of the results delivered by the different software pipelines for the analysis of metagenomics data.

Two additional points should be stressed. First, in this proficiency test dataset, we included African swine fever virus and Escherichia virus phiX174 sequences as within run contaminants, which only three and four participants, respectively, correctly assessed. Noteworthy, the same effect frequently occurs in real sequencing runs [Illumina, 2013 (between runs), Sinha et al., 2017; Illumina, 2017 (within run)]. Therefore, knowledge of the content of samples from the same and previous runs might be necessary to take into account, information that was not available for this proficiency test. Most importantly, however, awareness to the problem needs to be raised. Second, the interaction between the different sectors (human, veterinary, and food) and the disciplines within these, e.g., virology, bacteriology, parasitology, needs to be strengthened. This must necessarily include enhanced awareness of the pathogens of importance for other sectors and disciplines, including reporting to the respective colleagues in case relevant pathogens are detected.

## Data Availability Statement

The datasets presented in this study can be found in online repositories. The names of the repository/repositories and accession number(s) can be found below: data are available from https://www.ebi.ac.uk/ebisearch/overview.ebi/about under accession PRJEB37463.

## Author Contributions

DH and CW conceived the study, analyzed data, and wrote the manuscript. DH created the artificial dataset. JG, AB, JM, SMa, RE, CD, ST, IC, SMo, MJ, TP, RH, SP, ML, MH, AW, PC, LC, MT, JS, CS, AD, AN, and MB analyzed the artificial dataset and assessed the results. All authors revised and approved the manuscript.

## Conflict of Interest

The authors declare that the research was conducted in the absence of any commercial or financial relationships that could be construed as a potential conflict of interest.

## Footnotes

1 https://www.compare-europe.eu/library/protocols-and-sops

2 https://rstudio.com/

## Supplementary Material

Supplementary Figure 1 Specificities of read assignments calculated from the complete read set based on the species assignments. Sequences of the taxa labeled gray (*Brugia malayi*, *Caenorhabditis remanei*, *Danio rerio*, and *Scomber japonicus*) were downloaded unintentionally as part of the *Anisakis* sequence dataset.

Supplementary Figure 2 Correct classification rates of read assignments calculated from the complete read set based on the species assignments. Sequences of the taxa labeled gray (*Caenorhabditis remanei*, *Brugia malayi*, *Danio rerio*, and *Scomber japonicus*) were downloaded unintentionally as part of the *Anisakis* sequence dataset.

Supplementary Figure 3 Negative predictive values of read assignments calculated from the complete read set based on the species assignments. Sequences of the taxa labeled gray (*Caenorhabditis remanei*, *Brugia malayi*, *Danio rerio*, and *Scomber japonicus*) were downloaded unintentionally as part of the *Anisakis* sequence dataset.

Supplementary Materials and Methods Emails with requirements to participants and details on the calculation of sensitivity, specificity, correct classification rate, positive predictive value, and negative predictive value are presented.

Summary and Assessment of the Participants All summary and assessment files sent in by the participants are included.
