# Supplementary material for: Proficiency Testing of Metagenomics-Based Detection of Food-Borne Pathogens Using a Complex Artificial Sequencing Dataset
Source: Front Microbiol. 2020 Nov 4;11:575377. doi: 10.3389/fmicb.2020.575377 (PMC7672002; doi:10.3389/fmicb.2020.575377)
Supplement: Supplementary Figure 1 — Specificities of read assignments calculated from the complete read set based on the species assignments. Sequences of the taxa labeled gray (Brugia malayi, Caenorhabditis remanei, Danio rerio, and Scomber japonicus) were downloaded unintentionally as part of the Anisakis sequence dataset. [file Data_Sheet_1.pdf]

Supplementary Materials and Methods

Creating a synthetic metagenomics dataset

The synthetic metagenomics dataset was created using ART_Illumina, Q Version 2.5.8 (Huang et al., 2012) and a number of EST (expressed sequence tag) and coding sequence data retrieved from the NCBI database (see Table 1 in the main manuscript). It was designed as Illumina dataset sequenced with an Illumina MiSeq instrument using v3 chemistry in single end 250 bp mode (software call with art_illumina -ss MSv3 -i inputfile.fna -o outfile-prefix -l 250 -f 35 -s 10 -na), however, the read quality profiles generated by the software were in inverted order along the reads. The dataset resembled sequencing of a sample of contaminated trout (analogous to the contaminated salmon investigated in the wet lab part) analyzed by shotgun RNA sequencing. Sequences of the taxa *Brugia malayi, Caenorhabditis remanei, Dania rerio,* and *Scomber japonicus* were downloaded unintentionally as part of the *Anisakis* sequence dataset. This could not be reproduced; hence, the reason for this download remains unclear.

Data analyses

The submitted read assignment tables were analyzed using R and RStudio (R v3.6.2, R Core Team, 2019; RStudio v1.2.5033, https://rstudio.com/). To enable a robust comparison of the classification results submitted in the read assignment tables, all reported taxonomic entities were first adjusted to the NCBI taxonomy database names by replacing deviating names with the official names. Ambiguous classifications, like for instance “Proteus” fitting to the vertebrate genus “Proteus” (family Proteidae, order Caudata), and to the bacterial genus “Proteus” (family Morganellaceae, order Enterobacterales), were set to “Unknown”. Hence, only unambiguous read classifications were taken into further account. After consolidation of the names, the corresponding taxonomy IDs were determined. Then, the reported highest-level taxonomic rank was determined and the corresponding lower order ranks (genus, family, and superkingdom) were identified in the NCBI taxonomy database. Finally, all reads which were not included in the read assignment tables were assigned “not reported”. Thereafter, sensitivity, specificity, correct classification rate, positive predictive value, and negative predictive value were calculated from these read-to-taxon assignments based on genus and species assignments each. To this end, the assignments reported by the participants were compared with the known correct taxa of the reads in the artificial dataset to define the true positives (tp), the false positives (fp), the true negatives (tn), and the false negatives (fn).

From these classifications, the evaluations were calculated according to the following formulae:

sensitivity = tp / (tp + fn)

specificity = tn / (tn + fp)

correct classification rate = (tp + tn) / (tp + tn + fp + fn)

positive predictive value = tp / (tp + fp)

negative predictive value = tn / (tn + fn)

For the final rating of the participants' assessments, the expected assessments were defined as shown in Table 1 in the main manuscript. The submitted assessments were rated in five different classes: (i) species detected and rated by the participant as expected, (ii) species detected and rated more serious than expected, (iii) species detected but assessed less critical than expected, (iv) species detected but not assessed, and (v) species not detected (according to the submitted read assignment table).

References

Huang, W., Li, L., Myers, J. R., Marth, G. T. (2012). ART: a next-generation sequencing read simulator. *Bioinformatics* 28, 593–594. doi: 10.1093/bioinformatics/btr708

R Core Team. (2019). R: A language and environment for statistical computing. R Foundation for Statistical Computing, Vienna, Austria. URL https://www.R-project.org/.

Höper, Dirk

Von: Höper, Dirk
Gesendet: Mittwoch, 25. April 2018 10:23
An: Höper, Dirk
Cc: Wylezich, Claudia
Betreff: COMPARE Food Ring Trial - dry lab

Dear colleague

You registered as participant for the dry lab part of the COMPARE food metagenomics proficiency test. Therefore, you are soon going to receive a download link for the dataset to analyze. Please follow that link and enter foodPT2018 as password for the download.
The file contains a synthetic dataset mimicking an Illumina MiSeq shotgun RNA dataset obtained with MiSeq v3 chemistry with single end 250 bp sequencing. It was generated using ART_Illumina (Q Version 2.5.8; https://doi.org/10.1093/bioinformatics/btr708) and a number of different sequences from the NCBI databases as input. Following the wet lab part, the dataset contains data resembling a sequencing run of a sample of contaminated fish.
As indicated in the announcement, please conduct your bioinformatics analysis and upload the obtained results in the dedicated COMPARE data hub. In addition, we ask you assess your results and send us a summary of your assessment with regard to both potentially present pathogens and sequencing artefacts.
Please do not hesitate to contact me in case of further questions or any problems with the file!
On behalf of the COMPARE Food Ring Trial team
Best regards

Dirk Höper

Dr. Dirk Hoeper
Laborleiter
Institut für Virusdiagnostik

Friedrich-Loeffler-Institut
Bundesforschungsinstitut für Tiergesundheit
Federal Research Institute for Animal Health
Südufer 10 | 17493 Greifswald - Insel Riems
Tel: +49 38351 7 1235/1326 | Fax: +49 38351 7 4235
https://www.fli.de/

Höper, Dirk

Von: Höper, Dirk
Gesendet: Donnerstag, 17. Mai 2018 14:14
An: Höper, Dirk
Cc: Wylezich, Claudia
Betreff: AW: COMPARE Food Ring Trial - dry lab

Dear colleague

Since we had a few requests regarding the content and format of your result report, we would like to specify the procedure in more detail:

1. Please prepare a table in which you provide per read the taxonomic classification. This table should comprise the two columns “Read accession” and “Species”. Do not report genotype, subtype or strain level but the official species name. In case a read could not be classified, report “unknown” in the column “Species”. Upload the table here [redacted] Click on the “Provide” link and follow the instructions. Please enter [redacted] and [redacted] as “Recipients” in the upload dialogue.
2. Please prepare a report as Word file or pdf. In this report, compile the most important species you detected in a summary in table format. In this table, include all information that you deem relevant. Assess the results you compiled in the table and write a short text summarizing your assessment. Consider potentially present pathogens, sequencing artefacts, possible sequencing lab contaminations, and other important facts. Please send this summary by email to [redacted] and [redacted]

Please do not hesitate to contact us in case of further questions or any problems!
On behalf of the COMPARE Food Ring Trial team
Best regards

Dirk Höper

Dr. Dirk Hoeper
Laborleiter
Institut für Virusdiagnostik

Friedrich-Loeffler-Institut
Bundesforschungsinstitut für Tiergesundheit
Federal Research Institute for Animal Health
Südufer 10 | 17493 Greifswald - Insel Riems
Tel: +49 38351 7 1235/1326 | Fax: +49 38351 7 4235
https://www.fli.de/

Analysis of the dataset from COMPARE Food Metagenomics Ring Trial 2018

	% of classified reads	comments
**human pathogenic species**		
Escherichia coli	5,6	
Listeria monocytogenes	1,7	
Burkholderia pseudomallei	0,56	
Salmonella enterica	21	
Aspergillus flavus	0,35	
Norwalk virus	0,16	
Human mastadenovirus C	0,01	can cause Gastroenteritis dependent on serotype
**opportunistic pathogens**		
Bacteroides fragilis	3,4	microbiota
Corynebacterium kroppenstedtii	0,005	microbiota
Proteus mirabilis	0,002	microbiota
Mycobacterium colombiense	0,27	
Fusobacterium nucleatum	6,5	peridontal disease
**Fish pathogens**		
Atlantic salmon swim bladder sarcoma virus	0,0003	tumor inducing virus from salmon
Pseudomonas fluorescens	0,13	can cause fin rod in fish
Ichthyophthirius multifiliis	0,15	only 8 reads
**Human apathogenic species**		
Aspergillus foetidus dsRNA mycovirus	0,019	Virus from Aspergillus
Lactobacillus delbrueckii	3,5	
Lactobacillus acidophilus	30	
Cyberlindnera jadinii	0,01	Yeast, e.g. used as flavoring in processed foods
**Artefacts/ Contaminations**		
Escherichia virus M13	0,016	synthetic construct/helper Phage M13HT/Plant vector/Expression vector
Enterobacteria phage phiX174 sensu lato	0,008	PhiX, positive control
African swine fever virus	0,003	Lab contamination / Sequencing contamination

Quality assessment of the data with fastqc (Andrews, S. 2010) revealed bases of bad quality at the beginning of the reads. Therefore the reads were trimmed. For the taxonomic assignments of the reads to bacteria, parasites and fungi species, kraken with a custom database constructed from bacteria, virus, fungi and parasite genomes in RefSeq release 87 and MiniKraken DB were used. Additionally pathoLive (Tausch, S., unpublished) analysis was performed for the classification of viral reads. The kraken analysis with the custom database resulted in many false-positive results. Therefore results were confirmed with blastn.

Three foodbourne bacteria were detected in the dataset that can cause gastro enteric infections: *Salmonella enterica* and *Escherichia coli* and *Listeria monocytogenes*. Reads belonging to *Burkholderia pseudomallei* that can cause melioidosis when inhaled or ingested were identified. Furthermore *Norovirus* that cause gastro intestinal infection in humans and *Aspergillus flavus* that produces highly carcinogenic aflatoxins were detected. *Human mastadenovirus C* that can cause mild gastrointestinal infection in humans was detected in a very low abundance. The opportunistic *Mycobacterium colombiense* pathogen was found in a small abundance and can pose a health risk for immunocompromised humans. Further detected opportunistic bacteria that are part of the human microbiota and cause no harm when ingested are: *Bacteroides fragilis*, *Corynebacterium kroppenstedtii* and *Proteus mirabilis*. Sequences of the oral bacterium *Fusobacterium nucleatum* were identified that plays a role in periodontal disease. Detected bacteria species that cause no health risk for humans are *Lactobacillus acidophilus*, *Lactobacillus delbrueckii* and *Pseudomonas fluorescens*. The *Aspergillus foetidus dsRNA mycovirus* could be detected and hints towards the contamination of the salmon with Aspergillus which is in concordance with the detection of *Aspergillus flavus*. The detection of sequences of the *African swine fever virus* is probably a lab contamination or a bleed-through contamination resulting from sequencing a different sample containing *African swine fever virus* in the same or in a previous sequencing run. The reads classified as *Escherichia virus M13* probably originate from a synthetic construct like a vector or a helper phage. The sequences that were classified as *Enterobacteria phage phiX174 sensu lato* are an artefact originating from the PhiX positive control that is usually spiked in an Illumina sequencing run.

With the results of this analysis we advise against the consumption of this contaminated salmon.

FOOD COMPARE PROFICIENCY TEST

Methods

In order to identify the different species in the supplied shotgun RNA metagenomics sample 1x250, we followed three different approaches. Species identification is consistent across the methods as we will see later.

Quality analysis was done with FastQC, and trimming was performed to remove adapters, low-quality ends. Trimming was done with Trimmomatic removing those nucleotides in 3' which phred quality was lower than 10 or which an average of 15 in a four nucleotides window, plus removing all reads shorter than 50bp. This trimming step dropped 8137323 sequences (81.71%).

We would like to remark an unusual bad quality 5' end was observed at the 25 firsts bases of sequenced reads, that could not be removed with our trimming approaches.

1) Based on mapping and assembly approaches

The first method we used is **PikaVirus** (https://github.com/BU-ISCIII/PikaVirus), our in-development tool for metagenomics analysis, which follows a new mapping approach integrated with traditional assembly and blast annotation.
A mapping against each taxonomic genomic database to identify their taxons is performed. Host (Atlantic Salmon) mapping reads are also removed. Reads were assembled and blasted against their reference genomes database inside the corresponding taxon to identify species and percentage of the coverage of each genome. Assembled contigs are also combined into the output to help identifying the right organisms.

Bowtie2 was used for the mapping steps, and Spades for the assembly. Each resulting contig was matched against its reference genome with BLASTn and all results with an identity percentage below 90% were removed. Bedtools calculates the coverage of each genome by the assembled contigs.

PikaVirus reports five bacteria found in the sample, with the corresponding percentage of genome that was able to rebuild with the reads provided in the sample: Lactobacillus acidophilus (62%), Fusobacterium nucleatum (42%), Escherichia coli (40%), Salmonella enterica (23%), Arthrobacter sp (16%). It also reports a Norovirus (99%) and a Enterobacteria phage phiX174 (97%).

In a second approach, ***de novo* assembly** was carried by **oases** and then blasted the resulting transcripts against both the whole genome database and the ribosomal RNA sequence (SILVA) database. Mapping and blasting of the transcript were carried in an analogous form as in the paragraph above.

This method shows us the positive control that we did not remove this time: Atlantic Salmon and, by homology, Rainbow Trout. It also finds the Lactobacillus acidophilus, Salmonella enterica, Escherichia

coli, Fusobacterium nucleatum. For viruses, we found Norovirus and Enterobacteria phage phiX174.

2) Based on rRNA clustering

A different version of the second approach was carried using a dedicated bioinformatics pipeline to assign sequences at different taxonomic levels depending on their specificity called Meta-Total RNA-sequencing (MeTRS). This pipeline also uses the SILVA database to identify the species. The pipeline was modified to suit better our dataset, by replacing bowtie for bowtie2 which works better for the length of our sequences.

MeTRS only returns a non consistent hit, Sporosarcina_globispora. No viruses were found with this approach. It seems that this approach is not sensitive enough using SE reads.

Finally, as directly mapping reads for the fastq file to the genomic database is not a reliable method for identifying the right species for each read, a fourth analysis was done with kaiju. Kaiju is a program for the taxonomic classification of reads from whole-genome sequencing of metagenomic DNA. Reads are translated in six frames coding and assigned to taxa using the NCBI taxonomy and a non redundant reference database of protein sequences from eukariotic microbial, bacteria, archaea and viruses.

3) Based on protein identity analysis

Kaiju results (Table 2) identify some bacteria we already found with the other methods, but did not manage to identify the viruses species despite detecting them.

% Reads	# Reads	Specie
1.576601	27734	Lactobacillus acidophilus
1.204649	21191	Herbaspirillum rubrisubalbicans
0.929281	16347	Salmonella enterica
0.571201	10048	Monosiga brevicollis
0.562731	9899	Salpingoeca rosetta
0.272355	4791	Virus
76.093644	1338564	unclassified

Table 2. Reads corresponding to each specie.

Results

Combining the results of the previous described four methods (Table 1), we conclude that the species in the sample are **Lactobacillus acidophilus, Escherichia coli, Salmonella enterica, Norovirus and Enterobacteria phage phiX174**.

MeTRS results were excluded, as they do not agree at all with the other three methods and its result is not related with food poisoning bacteria. The rest of the methods agree on Lactobacillus acidophilus Salmonella enterica, and Escherichia coli was robustly identified by two methods. Norovirus and Enterobacteria phage phiX174 were also detected by those two methods, plus Kaiju predicted 3 viruses but could not identify them.

Method	Bacteria	Virus
PikaVirus	**Lactobacillus acidophilus**<br>Fusobacterium nucleatum<br>**Escherichia coli**<br>**Salmonella enterica**<br>Arthrobacter sp	**Norovirus**<br>**Enterobacteria phage phiX174**
Oases	**Lactobacillus acidophilus**<br>**Salmonella enterica**<br>**Escherichia coli**<br>Fusobacterium nucleatum	**Norovirus**<br>**Enterobacteria phage phiX174**
MeTRS	Sporosarcina globispora	-
Kaiju	**Lactobacillus acidophilus**<br>Herbaspirillum rubrisubalbicans<br>**Salmonella enterica**<br>Monosiga brevicollis<br>Salpingoeca rosetta	-

Table 1. Top species identified by each method.

Participant 3, Summary and Assessment, page 1 / 1

Most important microbial species detected

Salmonella enterica subsp. enterica serovar Typhi<br>(human pathogen not found in animals, indicative of human faecal contamination)
Escherichia coli O104:H4 with stx phage<br>(human pathogen, possibly indicative of human faecal contamination)
Burkholderia pseudomallei (agent of melioidosis, endemic in parts of southeast Asia, related to flooding)
Escherichia coli APEC O78<br>(avian E coli found on flies, indicative of faecal contamination)
Shigella flexneri<br>(human pathogen, possibly indicative of human faecal contamination)
Listeria monocytogenes<br>(human pathogen typically found on food, iften linked to food production chain)
Fusobacterium nucleatum<br>(bacteria normally found in oral cavity, vagina or colorectal cancel, faecal contamination)
Bacteroides fragilis<br>(normally found in human colon, phages are tracer of faecal contamination)
Candidatus Sulcia muelleri<br>(normally found in sap-feeding insects, not food)
Mycoplasma hyopneumoniae (species of bacteria known to cause the disease porcine enzootic pneumonia), unlikely to be found on salmon, faecal contamination?
Norovirus GV (murine norovirus, not a human pathogen)<br>Unlikely to be on food sample
Hepatitis C virus (human pathogen, but route of transmission is via blood), highly unlikely to be found on food sample, contamination with human blood?

Interpretation: many bacterial pathogens were detected in the fastq file, Listeria monocytogenes is the only “expected” species; Salmonella Typhi , Shigella, Burkholderia, E coli point towards a heavy sewage contamination of the salmon, probably of south east asian origin (B. Pseudomallei & S. Typhi). Hepatitis C is a strange finding as this virus not very stable outside humans and could be indicative of a lab contamination or human blood contamination.

Compare food PT (dry)

Description of procedure:
Fastq data was processed using MGmapper pipeline[1] (https://bitbucket.org/genomicepidemiology/mgmapper). Initially reads are mapped against a phiX174 reference sequence to remove potential control library reads. Next against the following databases: Bacteria, Bacteria_draft, HumanMicrobiome, Archaea, Virus, Fungi, Protozoa, Human, Vertebrates_other and nt.

In short reads were mapped to reference sequence databases using BWA mem (version 0.7.12)[2]. Only reads mapping with an alignment score >= 30 were considered. Read are annotated (in MGmapper program) at strain level and subsequently taxonomy is collapsed species level. Species annotations are provided if 4 criteria are fulfilled:

1. Minimum read count = 20
2. Maximum edit distance <= 15%
    a. Calculated as (sum of edit distances for reads assigned to a species)/(total number of nucleotides assigned to a species).
    b. The edit distance is the number of operations that is needed to perform to make a read sequence match the reference sequence. It is roughly the number of nucleotide mis-matches.
3. Uniquely mapped reads >= 0.5%
    a. A uniquely mapped read has an alignment score that is higher than any other alignments scores i.e. the read can be uniquely assigned to a single reference sequence.
4. Minimum Size normalized abundance >= 0.01
    a. Size normalized abundance = 100*(Number of reads mapped to a reference sequence)/(Size of reference sequence (bp)).

Table.1 Summary of top 5 most abundant species.

Species	% Reads	Reads	Reads uniq
Oncorhynchus mykiss	77.46	7714373	6424203
Oncorhynchus kisutch	6.70	667200	467299
Oncorhynchus tshawytscha	2.07	206324	163320
Lactobacillus acidophilus	1.76	174924	1519
Salmo salar	1.29	128888	79316

Table 2. Summary of Pathogens, sequencing artefacts and contaminations.
Note: Certain contaminations can be difficult to identify without control samples. For example, certain microorganisms may be part of the natural microbiome of fish or could have been introduced during sample handling and processing.

Species	% Reads	Reads	Reads uniq
Salmonella enterica	1.26	125163	860
Escherichia coli	0.91	90428	2844
Gallus gallus	0.62	61342	59485
Listeria monocytogenes	0.55	9904	175
Anisakis simplex	0.07	6596	5433
Homo sapiens	0.05	4630	3818
Aspergillus flavus	0.04	3781	2697

Mycobacterium colombiense	0.017	1688	1688
Norwalk virus	0.009	946	943
Caligus clemensi	0.008	792	766
Caligus rogercresseyi	0.002	191	75
synthetic construct	0.007	665	18
Ichthyophthirius multifiliis	0.001	124	124

Reads in fastq file: 9958736

References:

1 Petersen, T. N. *et al.* MGmapper: Reference based mapping and taxonomy annotation of metagenomics sequence reads. *PLoS One* **12**, e0176469 (2017).

2 Li, H. Aligning sequence reads, clone sequences and assembly contigs with BWA-MEM. https://arxiv.org/abs/1303.3997 (2013).

Food Metagenomics Ringtrial – dry lab

In the table below we present the summary of key bacteria and viruses observed in the synthetic dataset. The data was analysed using kraken (using the minikaraken database) and then filtered on the basis of the number of reads to detemine the species present. We used a threshold of 500 reads for this purpose. At this level some samples could not be definitively identified to the species level, and these are indicated in the table.

The presence of phiX is most likely a carry over from the sequencing lab. Those species for which there were less than 1000 reads would need further confirmation before we would release the information.

% reads	# reads	Identity
94.72	9432532	unclassified
4.99	497049	Bacteria
1.23	122767	*Salmonella enterica*
0.39	39036	*Escherichia coli*
0.01	984	*Pseudomonas* spp.
0.08	7925	*Burkholderia pseudomallei* group
1.75	174459	*Lactobacillus acidophilus*
0.10	9764	*Listeria monocytogenes*
0.40	39995	*Fusobacterium nucleatum*
0.20	19926	*Bacteroides fragilis*
0.01	693	*Mycobacterium avium* complex
0.06	5495	Viruses
0.00	476	Herpesviridae
0.01	946	Caliciviridae
0.01	735	Enterobacteria phage phiX174 sensu lato

COMPARE metagenomics ring trial 2018 – Dry lab report

Quality of the sequencing reads was assessed with FastQC. No adapters were detected in the reads. Quality trimming of the read was performed with Trimmomatic. Reads belonging to the host species (*Oncorhynchus mykiss*) were removed using BBmap (id threshold = 0.65). Taxonomic classification of the reads was obtained with Kraken (database version 13/10/2017).

Results

The results obtained with Kraken are presented in the following table. Kraken results were converted to relative abundance using Bracken.

Species	Relative abundance
Lactobacillus acidophilus	34.79
Salmonella enterica	30.706
Escherichia coli	12.905
Fusobacterium nucleatum	8.008
Lactobacillus delbrueckii	4.893
Bacteroides fragilis	3.99
Listeria monocytogenes	2.003
Burkholderia pseudomallei	1.578
Mycobacterium colombiense	0.306
Norwalk virus	0.185
Pseudomonas fluorescens	0.161
Enterobacteria phage phiX174 sensu lato	0.117
Pseudomonas putida	0.075
Others	0.283

Table 1: Species identified in the metagenomics sample at a relative abundance > 0.05%.

Summary

The sample appeared to be heavily contaminated (95%) with DNA from the presumed host species, *Oncorhynchus mykiss*.

Bacterial species from the expected microflora of a preserved seafood products were identified: Lactobacillus acidophilus, Lactobacillus delbrueckii, Pseudomonas fluorescens, .

Bacterial species from foodborne pathogens were identified in a high abundance, susceptible to be a threat for a human consumer: Salmonella enterica, Listeria monocytogenes. Viral species of foodborne pathogens were also identified: Norwalk virus.

Several bacterial species originating most likely from human contamination (either during preparation of the product or preparation of the sample for analysis) were identified: Escherichia coli, Fusobacterium nucleatum, Bacteroides fragilis, Burkholderia pseudomallei, Mycobacterium colombiense.

Other taxa identified with this analysis (Pseudomonas putida, Enterobacteria phage phiX174 sensu lato) are believed to be artefacts resulting from mis-attribution of the sequencing reads due to their phylogenetic proximity to other species present in the sample.

DRY LAB

**Species**	**˜reads**
Human Mastadenovirus C	34
Norovirus GV	527
Aspergillus foetidus mycovirus	54
African swine fever virus	1
Anisakis simplex	5200
Bacteroides fragiles	11361
Burkholderia pseudomallei	4306
Escherichia coli /O104:H4	49942
Salmonella enterica serovar Typhi	68786
Mycobacterium columbiense	351
Listeria monocytogenes	5247
Lactobacillus delbrueckii/johnsonii	13388
Lactobacillus acidophilus	98888
Fusobacterium nucleatum	23618
Aspergillus flavus	842

Several food-borne pathogens were detected in the dataset. **Viruses:** Pathogen of importance is **Norovirus GV**. Human **Adenovirus C** can also be related to gastrointestinal diseases. One read of **African swine fever virus** was detected but is possibly a result of sequencing contamination (virus origin FLI). The identification of **Aspergillus virus i**ndicates the presence of Aspergillus flavus. Also reads of the **Aspergillus flavus aflatoxin** biosynthesis cluster were identified.

A **nematode, Anisakis simplex** was identified, which is a parasite of the human gastrointestinal tract.

Several **bacteria** were identified. Important food-borne pathogens **are E. coli O1404:H4, Salmonella enterica** and **Listeria monocytogenes. Burkholderia pseudomallei** is a highly pathogenic bacterium but not related to food-borne outbreaks. Abundance of a cloning vector could be an artifact of sequencing reagents and preparation.

COMPARE Food Ring Trial 2018

1. **potentially present pathogens**
   - **The *Calicivirdae*/Norwalk virus reads indicate the presence of noroviruses in the sample. This is the most important entero-pathogenic virus in the analysed sample.**
   - The Mastadenovirus reads indicate the presence of a Mastadenovurs in the samples. Some Mastadenoviruses can induce gastroenteritis. Therefore, the presence within the sample should be further analysed/confirmed.

   - **Listeria monocyogenes reads (aroudn 10000) indicate the presence of a pathogenic Listeria species! (see also specific phage reads). This zoonotic agent should be further confirmed in the sample.**

   - **The Enterobacteriaceae reads indicate the precsence of a potentially pathogenic Salmonella species (salmonella enterica)** in the sample as well **as E. coli. This reads have to be further evaluated to analyse for pathogenic species.**

   - Bacillus sequences are indicative for the presence of a bacillus species in the sample. But this could be also probiotic bacilla in the food sample.

   - **The reads matching Anisakidae like Anisakis simplex have to be further tested. They could indicate the presence of a pathogenic nematode from fish.**

2. **sequencing artefacts**
   - mimiviridae reads might be from the reagents in the workflow (e.g. the cleaning columns)

3. **possible sequencing lab contaminations**

   - the ASFARviridae reads are most likely the consequence of a lab contamination with ASFV
   - some of the other viral reads with very low read numbers and higher indentity values have to be further checked for possible contaminations (also from the kit systems; e.g. the bacculovirus or poxvirus reads)
   - the detected pseudomonas sequences could be a primary or a secondary contamination of the sample
   - the ca. 8000 reads of Burkholderia pseudomallei are not typical for a food sample. This is a bacterium from soil infecting humans in Asia. Lab contamination? Sample from Asia? Fish from Asia?
   - The detected Mycobacteriaceae reads are most likely a lab contamination and are not food-related.

4. **other important facts**
   - the sample is from a salmon species

- the retrovirus reads are most likely from retroviruses of the animal species where the sample originated (salmon mykiss)
- the phage-specific reads have tob e further analysed, since the presence of Listeria-specific phages indicates the presence of Listera, bacteria which are highly relevant food contaminants. Also E.coli specific phages make it likely that E. coli is present in the sqeuenced food sample.
- The herpesvirus specific reads have to be further analysed. Since only very few reads of different herpesviruses are detected, there is no indication of a relevant herpesvirus in the sample. Also cross reactivities with conserved sequences cannot be excluded. Contamination?
- Probiotic Lactobacillus acidophilus is mainly probiotic detected and might be on the surface of the tested sample.
- Fusobacterium nucleatum reads were detected and are most likely a contaminant under anaerobic conditions or secondary contaminants
- Low frequency bacterial reads indicate low level contaminats of the food sample as well as bacteria present in the microbiom of the animal where the food sample comes from.
- Single reads for Staph. aureus are most likely contamination from humans dealing with the samples.
- Single Clostridium reads are also from contamianting materials or present in the original food source in very low quantities.
- The detection of Aspergillus reads corresponds very well with the related virus reads. There might be an Aspergillus contamination of the food sample.
- Beside the fish component the sample might also include some chicken and some additional food components

METHODS

Bioinformatics

Species-level classification of metagenomic reads was performed using Kraken [1]. Multi-locus sequence types (MLSTs) were reconstructed from metagenomic reads using MetaMLST [2]. Strain-level identification of foodborne pathogens was achieved using PanPhlAn [3].

RESULTS & COMMENTS

Sequencing results

Sequencing of the wet lab sample on the Illumina NextSeq 500 produced a total of 86,077,720 high quality reads.

Species-level analysis

The dry lab sample was dominated by *Lactobacillus acidophilus* (39.4%), but the following putative foodborne pathogens were also present at >1%: *Salmonella enterica* (27.8%), *Escherichia coli* (9.8%), and *Listeria monocytogenes* (2.2%).

Similarly, although the wet lab sample was dominated by *Propionibacterium freudenreichii* (40.2%), the following putative foodborne pathogens were also present at >1%: *Staphylococcus aureus* (10.5%), *S. enterica* (9.1%), *E. coli* (8.4%), and *Streptococcus suis* (1.7%).

Additionally, both samples contained *Fusobacterium nucleatum* and *Bacteroides fragilis* at >1%, and although neither species is typically associated with foodborne disease, they can each cause disease in humans.

Strain-level analysis

The tool MetaMLST was used to reconstruct multi-locus sequence types (STs) from the metagenomic reads. MetaMLST detected *S. enterica* ST1 in the dry lab sample, while it detected *S. aureus* ST8 in wet lab sample. No other known STs were detected. However, potentially novel *E. coli* STs were detected in both samples.

PanPhlAn was subsequently used for more in-depth characterisation of these strains. The *S. enterica* strain present in the dry lab sample was closely related to Ty21a, while that present in the wet lab sample was closely related to UK-1. The *E. coli* strain present in the dry lab sample was closely related to O104:H4, while that in the wet lab sample was closely related to KOEGE 44 (106a). The *S. aureus* strain detected in the wet lab sample was closely related to FDAARGOS_33. No *L. monocytogenes* or *S. suis* strains were detected in either sample be PanPhlAn.

Species	Wet (%)	Dry (%)
Lactobacillus acidophilus	0.000347814	39.36460727
Salmonella enterica	9.122243956	27.82614573
Escherichia coli	8.387378531	9.75958722
Fusobacterium nucleatum	4.718812077	9.012662791
Lactobacillus delbrueckii	4.35E-05	5.291784958
Bacteroides fragilis	20.4363455	4.491460502
Listeria monocytogenes	0.000391291	2.212834032
Streptococcus suis	1.77508966	0.00721013
Propionibacterium freudenreichii	40.1599335	0
Staphylococcus aureus	10.54497646	0
Other (<1%)	4.854437734	2.033707367

REFERENCES

1. Wood, D.E. and S.L. Salzberg, *Kraken: ultrafast metagenomic sequence classification using exact alignments.* Genome Biol, 2014. **15**(3): p. R46.
2. Zolfo, M., et al., *MetaMLST: multi-locus strain-level bacterial typing from metagenomic samples.* Nucleic Acids Research, 2016: p. gkw837.
3. Scholz, M., et al., *Strain-level microbial epidemiology and population genomics from shotgun metagenomics.* 2016. **13**(5): p. 435-8.

COMPARE Food Metagenomic Ring Trial

DRY LAB PART – REPORT

AIM:

To Compare Microorganisms identity and corresponding abundances in a food matrix tested by shotgun metagenomics sequencing using Bioinformatics data analysis.

MATERIALS AND METHODS:

- The Quality control (QC) Analysis for the given metagenome dry lab data (foodPT2018.fastq file) is done using FastQC tool - version: 0.11.7. The FastQC is used to check the quality of the sequencing data. A Html report is generated for visualizing the quality of raw data.
- The MGmapper 2.4 - MetaGenomics mapper (https://cge.cbs.dtu.dk/services/MGmapper/) tool from Centre for Genomic Epidemiology (CGE) is used for analysing the given data. MGmapper uses sequence similarity based method for mapping our raw reads with the databases. Data is analyzed in three steps:
  1. Pre-processing: The MGmapper uses cutadapt for trimming low quality reads and adapters. Default settings in tools is used for this step.
  2. Mapping to database: The mapping step involves mapping the reads to the reference databases. The mapping mode used is Single end and the databases used in best mode are bacteria, virus, protozoa (2, 8, 10) and in full mode is resfinder (6).
  3. Post-processing: We did not get any positive hits for the default settings, hence the parameters for clade level post-processing is changed as below.
     (Max mismatch ratio = 0.15, Min read count = 20)

RESULTS AND DISCUSSION:

The reads are mapped with the reference sequences from bacteria, virus and protozoa databases using MGmapper tool. The best top hits have been reported here which has maximum covered positions and highest reads mapped to the reference sequences. The important species obtained from this data analysis are tabulated in Table 1.

In particular, the Lactobacillus acidophilus, Fusobacterium nucleatum, Norovirus and Acanthamoeba mauritaniensis species has maximum number of reads mapped when compared to other species (Figure 1). A total of 20 (Bacteria), 10 (Virus) and 84 (Protozoa) species were obtained as positive hits that parsed the post-processing parameters. The other hits were discarded and reported as negative by MGmapper tool. The percentage for the various databases in bestmode and full mode is reported in Table 2.

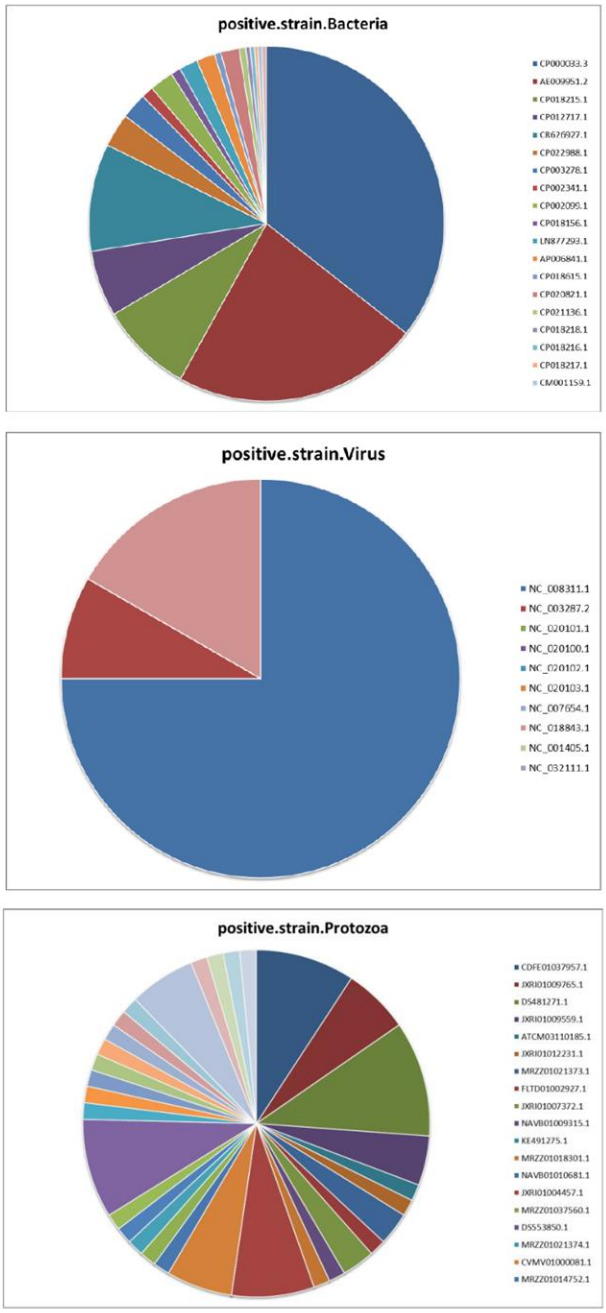

Figure 1: Graphical output showing different mapped species

Table 1: The MGmapper results

Database	Accession No	Covered Positions	Coverage	Reads	Species
**Bacteria**	CP000033.3	1596181	0.801	35824	**Lactobacillus acidophilus**
**Bacteria**	AE009951.2	1563798	0.719	22653	**Fusobacterium nucleatum**
**Bacteria**	CP018215.1	1134692	0.501	8453	**Lactobacillus delbrueckii**
**Bacteria**	CP012717.1	727416	0.318	6053	**Fusobacterium nucleatum**
**Bacteria**	CR626927.1	1688208	0.324	9818	**Bacteroides fragilis**
**Bacteria**	CP022988.1	531986	0.246	3123	**Lactobacillus delbrueckii**
**Bacteria**	CP003278.1	516691	0.108	2398	**Salmonella enterica**
**Bacteria**	CP002341.1	199637	0.094	1049	**Lactobacillus delbrueckii**
**Bacteria**	CP002099.1	506677	0.106	2233	**Salmonella enterica**
**Bacteria**	CP000033.3	178393	0.09	906	**Lactobacillus delbrueckii**
**Bacteria**	CP020821.1	395124	0.071	1688	**Mycobacterium colombiense**
**Virus**	NC_008311.1	7371	0.999	946	**Norovirus**
**Protozoa**	CDFE01037957.1	326	0.43	628	**Acanthamoeba mauritaniensis**
**Protozoa**	JXRI01009765.1	685	0.85	425	**Saccharina japonica**
**Protozoa**	DS481271.1	1388	0.862	673	**Plasmodium vivax**
**Protozoa**	JXRI01009559.1	587	0.694	269	**Saccharina japonica**
**Protozoa**	ATCM03110185.1	299	0.59	107	**Physarum polycephalum**

Table 2: The MGmapper overall statistics

Mapping mode	database	Percentage	Number of reads mapped
Fullmode	notPhiX	100	9958001
Fullmode	ResFinder	0.001	103
Bestmode	Bacteria	4.994	497303
Bestmode	Virus	0.015	1451
Bestmode	Protozoa	0.129	12797
-	Unmapped	94.863	9446450

OTHER OBSERVATIONS:

- The Per sequence GC content measured by FastQC is reported as failure since the GC content per read curve and the normal distribution curve varies (Figure 2).

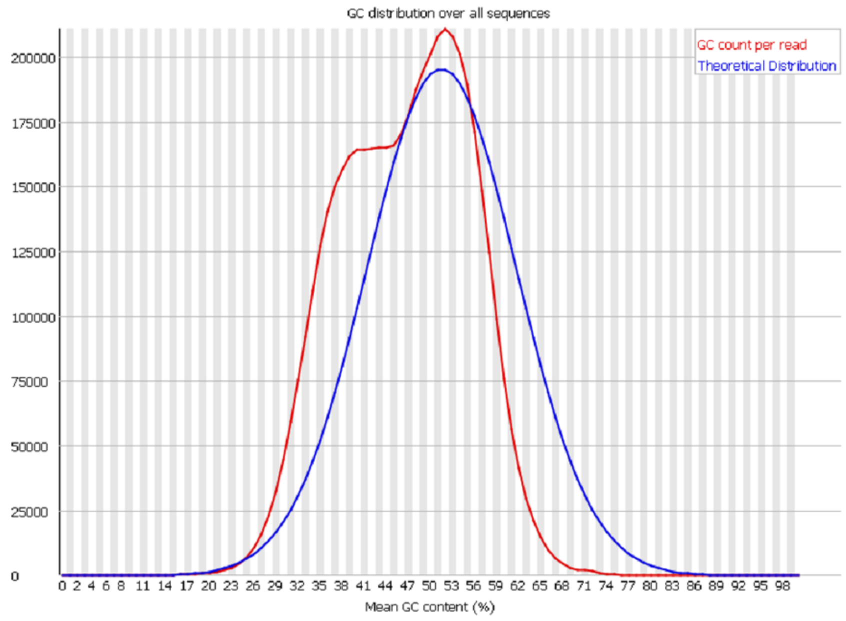

Figure 2: Per Sequence GC content graph obtained from FastQC

COMPARE Food Ring Trial – dry lab

Table. Report on most important species detected

	**Read accession**	**Species**	**E-value**	**Greatest length, bp**	**% identity**
1	LT905060	Salmonella enterica subsp. enterica serovar Typhi	0	10838	100
2	CP023535	Escherichia coli O104 strain FDAARGOS	0	6007	99,95
3	CP024245	Escherichia coli O27:H7 strain B4103-1	0	3815	99,92
4	CP023541	Escherichia coli O104:H21 str. CFSAN002236	0	3035	100
5	CP022461	Shigella sonnei strain 2015C-3807	0	1813	100
6	CP026098	Shigella flexneri strain FDAARGOS	0	1024	100
7	CP006046	Listeria monocytogenes J1-220	0	1348	100
8	CP025441	Listeria monocytogenes strain MF6172	0	1067	99,81
9	EF158010	Anisakis simplex	0	3257	100
10	DQ285629	Murine norovirus 1	0	7332	100

The most important species we detected, and which are potentially present as human pathogens are listed in 1-9. The selection is based on species which are known as human food borne pathogens, E-value, hit length and percentage identity. We also included Murine norovirus (10) in the table despite it is not human pathogen. We included this hit due to low E-value (0), contig 100% identity and a greatest hit length (7332 bp) that represents almost complete genome of Murine norovirus which is 7382 bp.

Species	% abundance	
Acanthamoeba castellanii	**0.13**	
Acanthamoeba comandoni	**0.11**	
Acanthamoeba lenticulata	**0.11**	
Acanthamoeba mauritaniensis	**0.11**	
Aspergillus flavus	**0.59**	
Aspergillus oryzae	**0.24**	
Bacteroides fragilis	**3.67**	
Balamuthia mandrillaris	**0.24**	
Botryosphaeria dothidea	**0.33**	
Burkholderia mallei	**0.19**	
Burkholderia pseudomallei	**1.28**	
Entomophthora muscae	**1.06**	
Escherichia coli	**16.57**	possible pathogen
Fusobacterium nucleatum	**7.31**	
Lactobacillus acidophilus	**32.23**	
Lactobacillus delbrueckii	**4.59**	
Listeria monocytogenes	**1.82**	possible pathogen
Mycobacterium colombiense	**0.31**	possible pathogen
Norwalk virus	**0.17**	possible pathogen
Pseudomonas fluorescens	**0.15**	
Salmonella enterica	**23.06**	possible pathogen
Trichophyton violaceum	**2.83**	
Unknown	**0.02**	

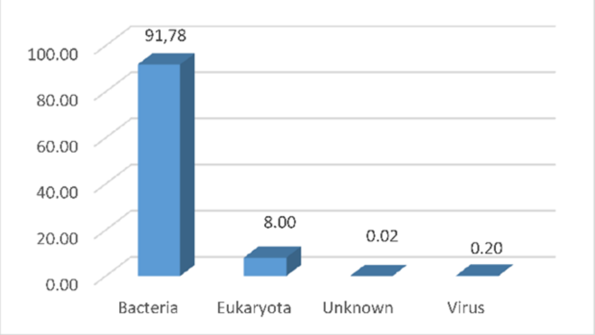

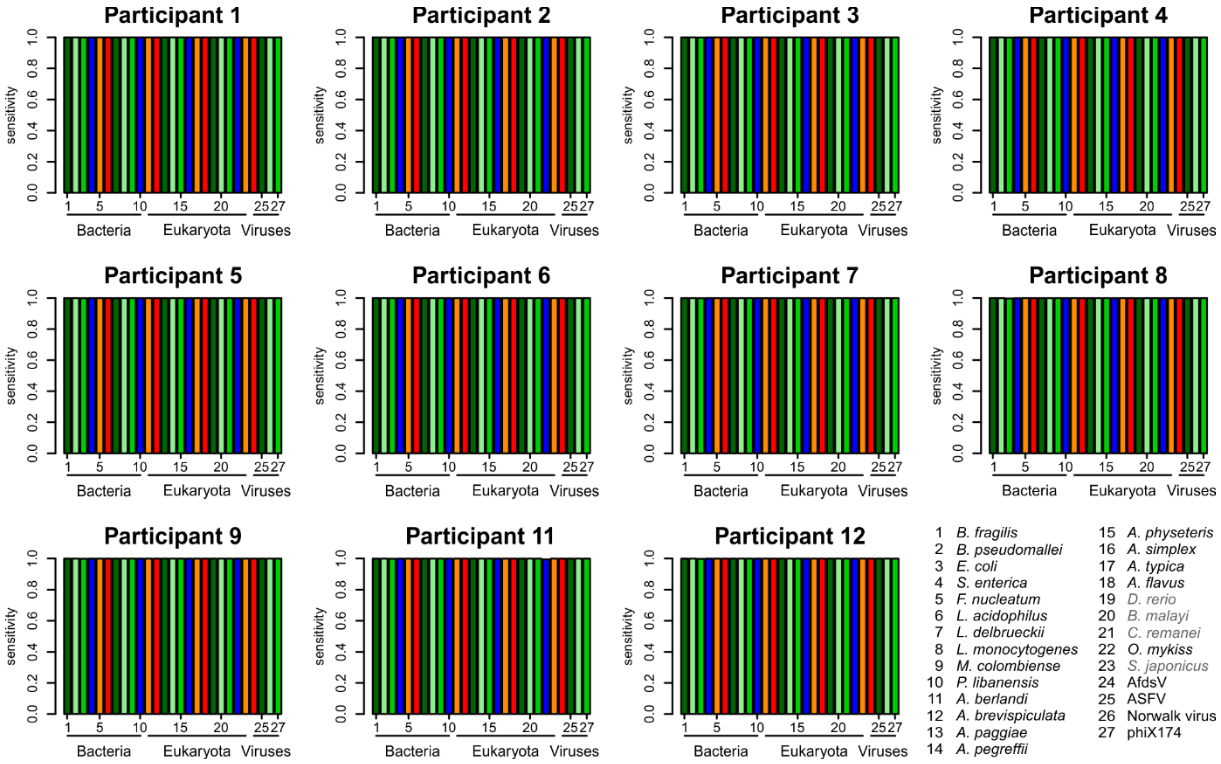

Supplementary Figure 1 Specificities of read assignments calculated from the complete read set based on the species assignments. Sequences of the taxa labelled grey (*Caenorhabditis remanei*, *Brugia malayi, Danio rerio,* and *Scomber japonicus*) were downloaded unintentionally as part of the *Anisakis* sequence dataset.

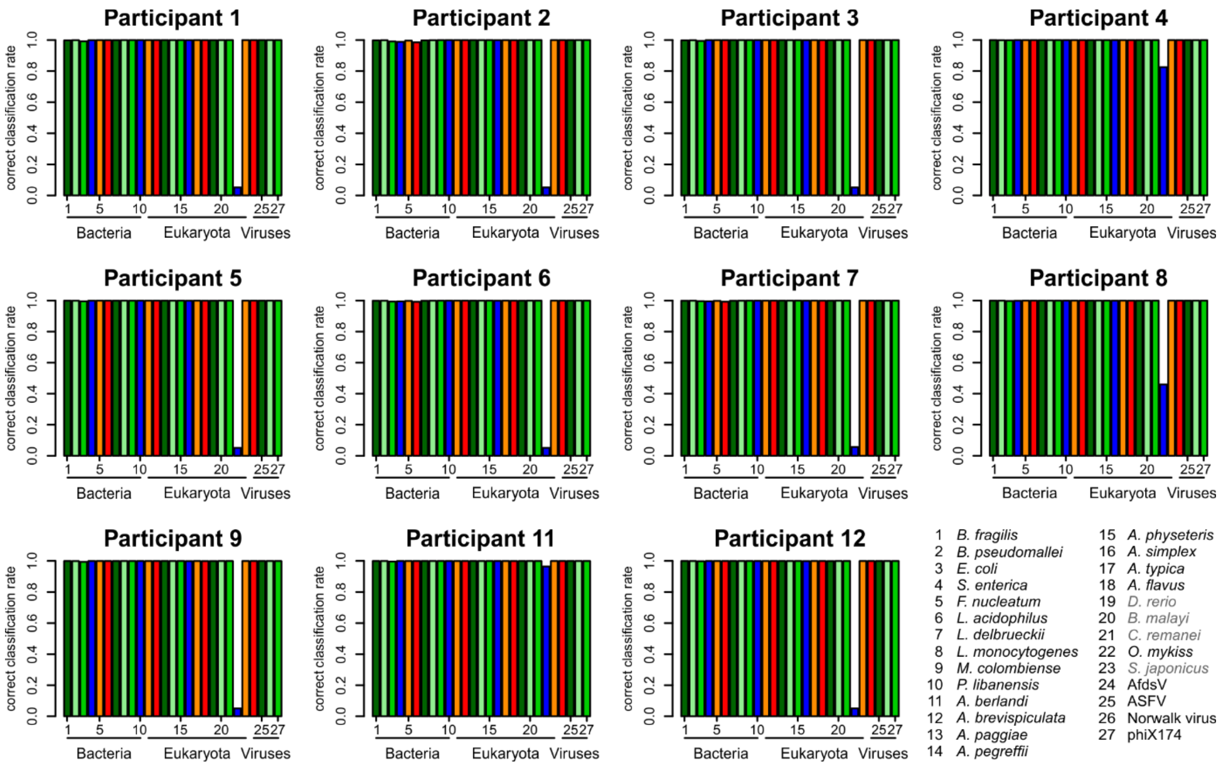

Supplementary Figure 2 Correct classification rates of read assignments calculated from the complete read set based on the species assignments. Sequences of the taxa labelled grey (*Caenorhabditis remanei*, *Brugia malayi*, *Danio rerio,* and *Scomber japonicus*) were downloaded unintentionally as part of the *Anisakis* sequence dataset.

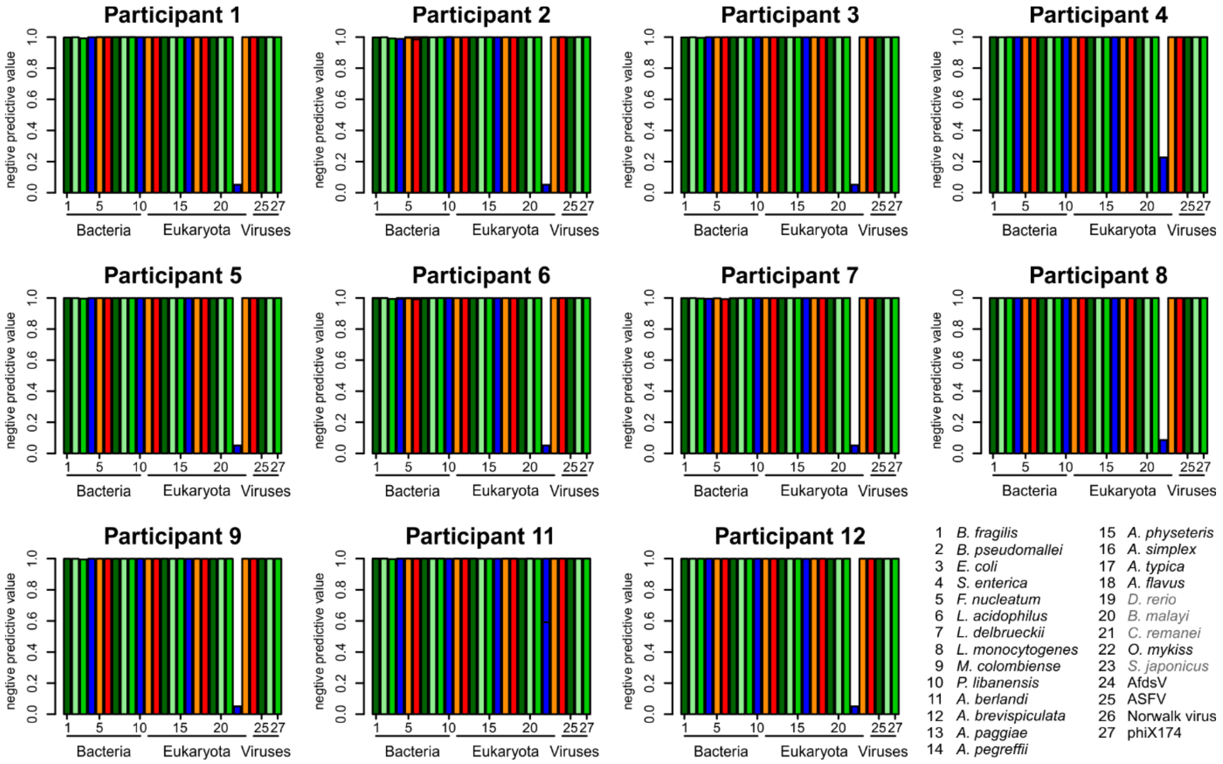

Supplementary Figure 3 Negative predictive values of read assignments calculated from the complete read set based on the species assignments. Sequences of the taxa labelled grey (*Caenorhabditis remanei*, *Brugia malayi*, *Danio rerio,* and *Scomber japonicus*) were downloaded unintentionally as part of the *Anisakis* sequence dataset.
